# Supplementary material for: Delayed diagnosis of congenital cataract in preterm infants: Findings from the IoLunder2 cohort study
Source: PLoS One. 2023 Aug 18;18(8):e0287658. doi: 10.1371/journal.pone.0287658 (PMC10437972; doi:10.1371/journal.pone.0287658)
Supplement: S1 Table — (DOCX) [file pone.0287658.s001.docx]

**S1 Table: Investigation of correlations between variables considered in analysis of outcome – bilateral cataract**

|  | White ethnicity | Deprivation | Prematurity | Non-ocular disorder | Ant segment dys | Whole globe anom | Family history ocular disease |
| --- | --- | --- | --- | --- | --- | --- | --- |
| Female sex | 0.22  p=0.6 | 0.32  p=0.6 | 0.48  p=0.5 | 0.50  p=0.5 | 0.30  p=0.6 | 0.69  p=0.4 | 3.29  p=0.07 |
| White ethnicity |  | 1.35  p=0.3 | 0.01  p=0.9 | 2.08  p=0.2 | 0.87  p=0.3 | 3.67  p=0.06 | 0.85  p=0.4 |
| Deprivation |  |  | **5.87**  **p=0.02** | 1.35  p=0.2 | 0.69  p=0.4 | 2.93  p=0.09 | 0.41  p=0.5 |
| Prematurity |  |  |  | **4.78**  **p=0.03** | 0.03  p=0.9 | 0.02  p=0.9 | 0.17  p=0.7 |
| Non-ocular disorder |  |  |  |  | 0.17  p=0.7 | 0.06  p=0.8 | **5.10**  **p=0.02** |
| Anterior Segment dysgenesis |  |  |  |  |  | 0.13  p=0.7 | 1.65  p=0.2 |
| Whole globe anomaly |  |  |  |  |  |  | **5.10**  **p=0.02** |
