# Supplementary material for: Delayed diagnosis of congenital cataract in preterm infants: Findings from the IoLunder2 cohort study
Source: PLoS One. 2023 Aug 18;18(8):e0287658. doi: 10.1371/journal.pone.0287658 (PMC10437972; doi:10.1371/journal.pone.0287658)
Supplement: S2 Table — (DOCX) [file pone.0287658.s002.docx]

**S2 Table: Investigation of correlations between variables considered in analysis of outcome – unilateral cataract**

|  | White ethnicity | Deprivation | Prematurity | Non-ocular disorder | Ant segment dys | Whole globe anom | Family history ocular disease |
| --- | --- | --- | --- | --- | --- | --- | --- |
| Female sex | 4.31  p=0.4 | 0.07  p=0.9 | 0.42  p=0.5 | 0.01  p=0.9 | 1.56  p=0.2 | 0.58  p=0.4 | 3.51  p=0.06 |
| White ethnicity |  | 0.22  p=0.6 | 0.89  p=0.3 | 0.42  p=0.5 | 0.57  p=0.4 | 1.32  p=0.3 | 0.21  p=0.7 |
| Deprivation |  |  | **3.34**  **p=0.05** | 3.30  p=0.07 | 1.39  p=0.2 | 0.06  p=0.8 | 0.44  p=0.5 |
| Prematurity |  |  |  | 0.12  p=0.7 | 0.21  p=0.7 | 0.28  p=0.6 | 0.62  p=0.6 |
| Non-ocular disorder |  |  |  |  | **18.6**  **p<0.001** | **6.22**  **p=0.01** | 1.41  p=0.2 |
| Anterior Segment dysgenesis |  |  |  |  |  | **20.7**  **p<0.001** | 0.22  p=0.6 |
| Whole globe anomaly |  |  |  |  |  |  | **5.2**  **p=0.02** |
