# Supplementary material for: Delayed diagnosis of congenital cataract in preterm infants: Findings from the IoLunder2 cohort study
Source: PLoS One. 2023 Aug 18;18(8):e0287658. doi: 10.1371/journal.pone.0287658 (PMC10437972; doi:10.1371/journal.pone.0287658)
Supplement: S2 Checklist — (DOCX) [file pone.0287658.s004.docx]

STROBE Statement—checklist of items that should be included in reports of observational studies

|  | Item No. | Recommendation | Page  No. | Relevant text from manuscript |
| --- | --- | --- | --- | --- |
| **Title and abstract** | 1 | (*a*) Indicate the study’s design with a commonly used term in the title or the abstract | 2 | “nested case-control study approach in a prospective population-based cohort study” |
|  |  | (*b*) Provide in the abstract an informative and balanced summary of what was done and what was found | 2 | “Of 186 children with congenital cataract, 17 children were born preterm (9%, gestational age range 24–37 weeks). Neonatal detection occurred in 64/186 (34%), and late detection in 64 children (34%). Late detection was independently associated with premature birth, specifically moderate/late preterm birth” |
| Introduction | | | |  |
| Background/rationale | 2 | Explain the scientific background and rationale for the investigation being reported | 5 | Recently, investigators have reported delayed vaccination amongst children born pre term.9,10 In this study, we compared the rates of delayed detection of congenital cataract among preterm and term/post term infants diagnosed with visually impactful cataract. |
| Objectives | 3 | State specific objectives, including any prespecified hypotheses | 5 | We hypothesized that preterm infants would have higher rates of delayed detection compared with term/post-term infants |
| Methods | | | |  |
| Study design | 4 | Present key elements of study design early in the paper | 5 | secondary analysis of data using a nested case control study approach in a prospective population-based cohort study |
| Setting | 5 | Describe the setting, locations, and relevant dates, including periods of recruitment, exposure, follow-up, and data collection | 5 | active surveillance methods and a national clinical research network to identify children undergoing cataract surgery during the first two years of life in UK and Ireland between January 2009 and December 2010. |
| Participants | 6 | (*a*) *Cohort study*—Give the eligibility criteria, and the sources and methods of selection of participants. Describe methods of follow-up  *Case-control study*—Give the eligibility criteria, and the sources and methods of case ascertainment and control selection. Give the rationale for the choice of cases and controls  *Cross-sectional study*—Give the eligibility criteria, and the sources and methods of selection of participants | 5 | all those children aged under 2 diagnosed with cataract requiring surgical intervention in the UK between January 2009 and December 2010 detailed clinical and demographic data were collected using standardised study specific collection instruments pre, per and post-operatively, with data collection from recruitment |
|  |  | (*b*) *Cohort study*—For matched studies, give matching criteria and number of exposed and unexposed  *Case-control study*—For matched studies, give matching criteria and the number of controls per case | No matching |  |
| Variables | 7 | Clearly define all outcomes, exposures, predictors, potential confounders, and effect modifiers. Give diagnostic criteria, if applicable | 6 | Congenital ocular anomalies were defined as structural malformations affecting the whole globe or specific components (ICD 10 codes Q11, 12, 14 and 15). ‘Truly’ congenital (rather than possibly infantile onset) cataract was defined as lens opacity co-existent with other congenital structural anomalies (eg persistence of fetal vasculature, anterior segment dysgenesis, whole globe anomalies, or congenital anomalies of lens shape).  non-white ethnicity, socio-economic deprivation status (index of multiple deprivation score within the lowest national quantile), the presence of anterior segment malformations or anomalies involving the whole globe (which may impact on ease of detection of ocular anomaly by non-ophthalmic clinicians) and the presence of a systemic impairment or disorder |
| Data sources/ measurement | 8* | For each variable of interest, give sources of data and details of methods of assessment (measurement). Describe comparability of assessment methods if there is more than one group | *6* | Child whose ‘truly’ congenital cataract was detected at either the neonatal eye health screening check, or at the six to eight week health check was considered to have been detected by the national screening programme.14 Any child detected after eight weeks of post-natal age was considered to have been detected ‘late’. |
| Bias | 9 | Describe any efforts to address potential sources of bias | 5  5  5 | population-based cohort study  active surveillance methods  prospectively collected standardised dataset for the nationally representative cohort |
| Study size | 10 | Explain how the study size was arrived at |  | n/a |

Continued on next page

| Quantitative variables | 11 | Explain how quantitative variables were handled in the analyses. If applicable, describe which groupings were chosen and why | 6 | Categorical data used: non-white ethnicity, socio-economic deprivation status (index of multiple deprivation score within the lowest national quantile), the presence of anterior segment malformations or anomalies involving the whole globe (which may impact on ease of detection of ocular anomaly by non-ophthalmic clinicians) and the presence of a systemic impairment or disorder |
| --- | --- | --- | --- | --- |
| Statistical methods | 12 | (*a*) Describe all statistical methods, including those used to control for confounding | 7 | Correlation between variables was investigated using χ2 tests (supplementary table) adhering to the current conventional threshold of p<0.05 for a statistically significant correlation. Multivariable analysis, using backward stepwise regression, included those variables with a p value of <0.10 in initial univariable analysis. We retained factors in the multivariable model if they altered the odds ratio estimate by more than 10% or were independently associated at a 5% significance level. |
|  |  | (*b*) Describe any methods used to examine subgroups and interactions |  | n/a |
|  |  | (*c*) Explain how missing data were addressed |  | n/a |
|  |  | (*d*) *Cohort study*—If applicable, explain how loss to follow-up was addressed  *Case-control study*—If applicable, explain how matching of cases and controls was addressed  *Cross-sectional study*—If applicable, describe analytical methods taking account of sampling strategy |  | n/a (nested case-control study) no matching |
|  |  | (*e*) Describe any sensitivity analyses |  | n/a |
| Results | | | | |
| Participants | 13* | (a) Report numbers of individuals at each stage of study—eg numbers potentially eligible, examined for eligibility, confirmed eligible, included in the study, completing follow-up, and analysed | 7 | The IoLunder2 study recruited 254 children undergoing cataract surgery under the age of two years, of whom 186 (73%) had truly congenital cataract. In 64 of these 186 children (34%) cataract was detected at the NIPE neonatal examination, and in a total of 58 children (66%) congenital cataract was detected by 8 weeks of age, as shown in Table 1 |
|  |  | (b) Give reasons for non-participation at each stage |  | n/a |
|  |  | (c) Consider use of a flow diagram |  | n/a |
| Descriptive data | 14* | (a) Give characteristics of study participants (eg demographic, clinical, social) and information on exposures and potential confounders | 8 | Table 2 |
|  |  | (b) Indicate number of participants with missing data for each variable of interest | 8 | Table 2 |
|  |  | (c) *Cohort study*—Summarise follow-up time (eg, average and total amount) |  |  |
| Outcome data | 15* | *Cohort study*—Report numbers of outcome events or summary measures over time |  |  |
|  |  | *Case-control study—*Report numbers in each exposure category, or summary measures of exposure | *8* | *Table 2* |
|  |  | *Cross-sectional study—*Report numbers of outcome events or summary measures |  |  |
| Main results | 16 | (*a*) Give unadjusted estimates and, if applicable, confounder-adjusted estimates and their precision (eg, 95% confidence interval). Make clear which confounders were adjusted for and why they were included | 12 | Table 4 |
|  |  | (*b*) Report category boundaries when continuous variables were categorized |  |  |
|  |  | (*c*) If relevant, consider translating estimates of relative risk into absolute risk for a meaningful time period |  |  |

Continued on next page

| Other analyses | 17 | Report other analyses done—eg analyses of subgroups and interactions, and sensitivity analyses |  |  |
| --- | --- | --- | --- | --- |
| Discussion | | | | |
| Key results | 18 | Summarise key results with reference to study objectives | 12 | In a population-based cohort study of children requiring surgery for cataract, we found a third of those with truly congenital disease (ie present at birth), were not detected via the national screening programme to detect cataract. Preterm birth was independently associated with late detection. |
| Limitations | 19 | Discuss limitations of the study, taking into account sources of potential bias or imprecision. Discuss both direction and magnitude of any potential bias | 12  13 | IoLunder2 is missing data on detection pathways for children with visually insignificant cataract (ie where surgery was not necessary), or those with severe ocular anomaly or systemic disease where visual prognosis was too poor for other reasons to support surgical intervention  Another possible limitation of the study is that it captures outcomes from the neonatal screening programme as implemented more than a decade ago. However, screening processes have not changed since this study was undertaken |
| Interpretation | 20 | Give a cautious overall interpretation of results considering objectives, limitations, multiplicity of analyses, results from similar studies, and other relevant evidence | 15 | Our findings identify important scope for improvement in the delivery of the neonatal eye screening programme, and possible necessary improvements in other routine or recommended early life public health processes for children born preterm. Attention is warranted to the training of non-expert examiners undertaking a challenging clinical examination. Improved communication of the findings of diagnostic examinations to referring bodies is needed, as well as improved data reporting systems for the outcome of screening processes, in order to create a closed audit loop. Further is needed on the clinical effectiveness of innovative screening tools for congenital cataract, and the child, family and care structure or system level challenges and opportunities for the implementation of whole population child health interventions aimed at improving visual outcomes for children born preterm. |
| Generalisability | 21 | Discuss the generalisability (external validity) of the study results | 13  14 | The IoLunder2 dataset remains the only nationally representative dataset with which to examine the factors affecting the detection of visually significant congenital and infantile cataract.  Investigations have reported under- or delayed vaccination of preterm infants versus their term-born peers – ie, challenges delivering necessary whole population interventions to these children within the recommended schedule |
| Other information | |  | | |
| Funding | 22 | Give the source of funding and the role of the funders for the present study and, if applicable, for the original study on which the present article is based | 7 | Role of the funding source  The funders had no role in study design, data analysis, data interpretation, or writing of the report. |

*Give information separately for cases and controls in case-control studies and, if applicable, for exposed and unexposed groups in cohort and cross-sectional studies.

**Note:** An Explanation and Elaboration article discusses each checklist item and gives methodological background and published examples of transparent reporting. The STROBE checklist is best used in conjunction with this article (freely available on the Web sites of PLoS Medicine at http://www.plosmedicine.org/, Annals of Internal Medicine at http://www.annals.org/, and Epidemiology at http://www.epidem.com/). Information on the STROBE Initiative is available at www.strobe-statement.org.
