# Supplementary material for: Delayed diagnosis of congenital cataract in preterm infants: Findings from the IoLunder2 cohort study
Source: PLoS One. 2023 Aug 18;18(8):e0287658. doi: 10.1371/journal.pone.0287658 (PMC10437972; doi:10.1371/journal.pone.0287658)
Supplement: Attachment — Submitted filename: comments .docx [file pone.0287658.s006.docx]

Delayed diagnosis of congenital cataract in preterm children: findings from the IoLunder2 cohort study

Comments:

The external validity of the research findings is questionable in the current research as it couldn’t be generalized to the population currently . the data obtained from 2010 surveillance. The situation might be totally differe nowadays.

The sample size in the current study is not justifiable as it include only those retrived by the surviellance system (the study might be underpowered)

The authors mentioned that they retain variables in the regression model with a P value <0.10 (is their any reference for that?)

In the tables, the percentage is aways approximated (the total is not 100%) in all tables

Table 1 is highly misleading and difficult to be understood also the median age at the time of diagnosis is not seen in the table however it is important (we can use in this table the terms delayed or timely detection)

Table 2: no need for a row to represeent missing cases (we can add if any as a footnote)

Table 2 doesn’t show a test of significance to reveal the difference between groups (where are the results of bivariate analysis). “minority ethnic group, residence in areas of socioeconomic, deprivation, premature birth or significant systemic diagnoses or impairments were common findings”……this is totally unaccepted . we rely on hypothesis testing”. So you need to add that there is no statistically significant difference……morover at the p value column to the table for better visualizing the results

Did you exclude those with missing gestational age from the total or included (in table 2 ,item 9)

Table 2 includes much information, it can be divided ito 2 tables. Also the visual acuity is not a risk factor for delayed detection, it is an outcome, I think it should be better presented even in a separate table with a clear test of significance. In the same point ,u can separate the simple range from the interquartile to be better understood)

Table 3: what is meant by univariate regression analysis, I think this is a mistake .this is just univariate analysis (using chi square test with estimation of risk)not regression analysis???

authors can combine the data in table 2 and 3 as stratification into unilateral and bilateral cataract didn’t add a lot.

In table 4, the multivariate analysis ,just add the significance of the whole model and overall significance

No comparisons in the discussion between results of the current work and other similar studies.
