# Supplementary material for: Delayed diagnosis of congenital cataract in preterm infants: Findings from the IoLunder2 cohort study
Source: PLoS One. 2023 Aug 18;18(8):e0287658. doi: 10.1371/journal.pone.0287658 (PMC10437972; doi:10.1371/journal.pone.0287658)
Supplement: Attachment — Submitted filename: Response to Reviewers.docx [file pone.0287658.s007.docx]

We thank the reviewers and editors for their comments. A point by point response from the authors is below:

| Editor / reviewer comment or query: | Author’s response | Location of change (in tracked version of manuscript) |
| --- | --- | --- |
| When submitting your revision, we need you to address these additional requirements.  1. Please ensure that your manuscript meets PLOS ONE's style requirements, including those for file naming. | We have amended the manuscript in line with the PLOS ONE's style requirements | Title page and text |
| 2. We note that the grant information you provided in the ‘Funding Information’ and ‘Financial Disclosure’ sections do not match.  When you resubmit, please ensure that you provide the correct grant numbers for the awards you received for your study in the ‘Funding Information’ section. | We have ensured that this information is consistent |  |
| 3. We note that you have indicated that data from this study are available upon request. PLOS only allows data to be available upon request if there are legal or ethical restrictions on sharing data publicly.  In your revised cover letter, please address the following prompts:  a) If there are ethical or legal restrictions on sharing a de-identified data set, please explain them in detail (e.g., data contain potentially sensitive information, data are owned by a third-party organization, etc.) and who has imposed them (e.g., an ethics committee). Please also provide contact information for a data access committee, ethics committee, or other institutional body to which data requests may be sent. | We have specified:  “Due to the potentially identifiable nature of the dataset and as participants have not given the necessary consent, the authors are prevented from publicly sharing individual level data under the ethics approval for this study from the National Health Service Health Research Authority National Research Ethics Service London – Harrow Committee (Research ethics reference 08/H0714/65). New ethics approval would be needed to either access individual level data without participant consent, or to re-approach participants in order to seek consent for data sharing. For more information, contact the authors. “ | Line 174-180 |
| 4. One of the noted authors is a group or consortium “British Congenital Cataract Interest Group”. In addition to naming the author group, please list the individual authors and affiliations within this group in the acknowledgments section of your manuscript. Please also indicate clearly a lead author for this group along with a contact email address.’ | This has been done | Acknowledgements |
| Reviewer #1 |  |  |
| “I believe the outcome of the current study will focus the light on an important health problem affecting the late preterm infants.” | We thank the reviewer for their comments |  |
| Line 81  The sentence needs revision | Reworded | Line 87-89 |
| Line 85  This sentence needs revision | This has been amended | 91-95 |
| Line 170  The sentence does not give a clear meaning. Please make it more comprehensive. | This has been amended | 186 - 191 |
| Line 171  The written results do not match with Table 1 | We have rewritten this section for clarity | 186 - 191 |
| Line 171 to 173  The sentence needs revision to be more comprehensive to the reader | This has been amended | 186- 191 |
| Table 1  I believe the table heading needs revision. The table summarizes the numbers and percentages of infants with congenital cataract detected at different phases | Amended to Number and proportions of children with congenital cataract detected at the different screening stages | Table 3 |
| Table 1  I don't believe that there is a value to calculate the 95% confidence interval here. | We disagree: it allows the reader a better understanding of the likely uncertainty around the reported proportions from different groups | No changes made |
| Line 180  "children" is a very general wrd, please make it more specific | Changed to ‘infants’ | Line 244 |
| Line 183  the variable was described as (Multi-system disorder / impairment) in Table 2, please try to use the same term in the text | For clarity we have used the term ‘non-ocular disorder / impairment’ | Text |
| Table 2  Additional information about the commonly associated ocular abnormalities should be mentioned if available | These have been provided | Lines 233 – 238 |
| Reviewer #2 |  |  |
| The external validity of the research findings is questionable in the current research as it couldn’t be generalized to the population currently . the data obtained from 2010 surveillance. The situation might be totally differe nowadays. | We disagree that our findings have limited applicability now as it seems unlikely that the situation regarding screening could be totally different now, other than the situation being considerably worse due to the negative impact of the pandemic on the delivery of preventative medicine globally.  Within the UK, the NIPE programme has been reviewed twice in the past decade (the authors are advisers to the UK National Screening Committee on the eye component of the NIPE) and no aspect of the NIPE programme content re eyes has changed ie timing, training of staff undertaking the examinations or content of the examination since 2010. We are not aware of any changes to existing mature screening programmes in other countries which would impede the generalisability of our findings to those settings. | No change |
| :  The sample size in the current study is not justifiable as it include only those retrived by the surviellance system (the study might be underpowered) | We thank the reviewer for giving us chance to describe the power of the IoLunder2 cohort as a nationally representative dataset. As we set out in lines 126 – 128, and as we had reported in an earlier paper (Solebo et al, ref 12 in this manuscript) our active surveillance approach ascertained **all** cases of cataract surgery in a child under 2 years within the surveillance window in the country. This was shown through comparison with the National Health Services’ data warehouse. | No change |
| The authors mentioned that they retain variables in the regression model with a P value <0.10 (is their any reference for that?) | Thank you for the comment. It is routine practice to use this approach to building a multivariable model (for example see <https://www.stata.com/manuals/rstepwise.pdf>)  We have not referenced this as we do not consider it necessary, just as we have not have referenced the p value threshold of 0.05 for statistical significance. | No change |
| In the tables, the percentage is aways approximated (the total is not 100%) in all tables | We have avoided the use of decimal points to aid legibility. | No change |
| Table 1 is highly misleading and difficult to be understood also the median age at the time of diagnosis is not seen in the table however it is important (we can use in this table the terms delayed or timely detection) | We note the confusion caused by table 1 and have amended this for clarity | Table 1 |
| Table 2: no need for a row to represeent missing cases (we can add if any as a footnote) | Amended | Table 2 |
| Table 2 doesn’t show a test of significance to reveal the difference between groups (where are the results of bivariate analysis). “minority ethnic group, residence in areas of socioeconomic, deprivation, premature birth or significant systemic diagnoses or impairments were common findings”……this is totally unaccepted . we rely on hypothesis testing”. So you need to add that there is no statistically significant difference……morover at the p value column to the table for better visualizing the results | The reviewer has we think assumed that this sentence on patient characteristics being common referred to them being MORE common in one group versus another. The sentence to which the reviewer refers is a descriptive analysis statement: hypothesis testing is not appropriate here. In order to avoid confusion we have stated that children with these characteristics are ‘represented’ in our cohort. | Line 203 – 205 |
| Did you exclude those with missing gestational age from the total or included (in table 2 ,item 9) | We have been explicit about the extent of missing data on gestational age in the total | Table 2 |
| Table 2 includes much information, it can be divided ito 2 tables. Also the visual acuity is not a risk factor for delayed detection, it is an outcome, I think it should be better presented even in a separate table with a clear test of significance. In the same point ,u can separate the simple range from the interquartile to be better understood) | We suggest that the flow of the article is better served by having these patient characteristics all presented in one table. It will, we think, be clear to the reader that we are not suggesting that all of these characteristics are being considered as risk factors | No change |
| Table 3: what is meant by univariate regression analysis, I think this is a mistake .this is just univariate analysis (using chi square test with estimation of risk)not regression analysis??? | Univariable logistic regression is the standard starting point in any statistical analysis that proceeds to multivariable analysis - as described for example in Wallisch C et al. (2022) Review of guidance papers on regression modeling in statistical series of medical journals. PLoS ONE 17(1): e0262918. <https://doi.org/10.1371/journal.pone.0262918>) | No change |
| authors can combine the data in table 2 and 3 as stratification into unilateral and bilateral cataract didn’t add a lot. | We have continued to keep these tables separate as there is clinical importance to meaningful differences between detection of bilateral and unilateral cataract. Firstly it has been suggested that unilateral cataract may be easier to detect as the examiner has a healthy contralateral eye for comparison. Secondly differences in outcomes between unilateral and bilateral cataract are well established. | No change |
| In table 4, the multivariate analysis ,just add the significance of the whole model and overall significance | We think that the reviewer is referring here to the multivariable analysis we have undertaken. We have taken the appropriate approach to reporting the findings from a multivariable regression model. | No change |
| No comparisons in the discussion between results of the current work and other similar studies. | We welcome any suggestions the reviewer may have on citable work on our hypothesis (that preterm infants have higher rates of delayed cataract detection compared with term/post-term infants). We have struggled to find similar studies and have concluded that our findings are novel. We have however contextualised our findings using work from other teams on delays in delivering other public health interventions, specifically vaccination, in preterm infants. These are discussed in the manuscript (references 9 and 10). | No change |
| moreover, no mentioned limitations for the presented work | The reviewer has missed our discussion of limitations in the second paragraph of the paper | Line 276 - 288 |
